# Vaginal Ligation of the Uterine Arteries for Uterine Fibromata

**Published:** 1897-03

**Authors:** 


### VAGINAL LIGATION OF THE UTERINE ARTERIES FOR UTERINE FIBROMATA.

Dr. Augustin H. Goelet, Professor of Gynecology in the New York School of Clinical Medicine, in a paper presented to the New York Obstetrical Society (*Am. and Obs. Journal*), believes that this operation, to be successful, should be done only in carefully selected cases, and that the uterine arteries should be divided, instead of simply being ligated, to secure complete obliteration of the vessels which supply the tumor with nourishment. Simple ligation is not considered sufficient where the tissues of the broad ligament are included in the ligature. When the vessel is not isolated, the ligature does not always rupture its coats, which is essential for complete obliteration. Shrinkage of the included tissues occurs from compression of the ligature, which loosens and the circulation through the vessel is often restored.

The operation should be limited to interstitial growths which do not extend above the level of the umbilicus, and small subperitoneal growths which spring from the uterine wall below the funuds, and where extensive adhesions with adjacent organs have not formed, through which the tumor might receive nourishment.

In those cases of the author, where the arteries have been divided, the result has been most satisfactory; that is, hemorrhage has been permanently controlled, menstruation has become normal, the other symptoms have disappeared, and the tumor has atrophied to such a degree that it could not be detected by careful bimanual examination; the uterus reducing to normal size.

The chief advantages in favor of this operation, in properly selected cases, may be enumerated as follows, viz.:

1. It is devoid of risk and the peritoneal cavity is not opened.
2. It is easily done.
3. It confines the patient to bed for two weeks only.
4. It removes all the symptoms produced by the tumor.
5. It effects marked diminution in the size of the tumor which, in some instances at least, entirely disappears.
6. It does not in any way interfere with a hysterectomy, should it subsequently become necessary.
7. It does not unsex or mutilate the patient.
8. The result is manifest within six months, and the patient is not disabled or inconvenienced by the operation.

